# Photodriven Transient Picosecond Top‐Layer Semiconductor to Metal Phase‐Transition in p‐Doped Molybdenum Disulfide

**DOI:** 10.1002/adma.202006957

**Published:** 2021-03-04

**Authors:** Nomi L. A. N. Sorgenfrei, Erika Giangrisostomi, Raphael M. Jay, Danilo Kühn, Stefan Neppl, Ruslan Ovsyannikov, Hikmet Sezen, Svante Svensson, Alexander Föhlisch

**Affiliations:** ^1^ Institut für Physik und Astronomie Universität Potsdam Karl‐Liebknecht‐Straße 24/25 14476 Potsdam Germany; ^2^ Institut für Methoden und Instrumentierung der Forschung mit Synchrotronstrahlung Helmholtz‐Zentrum Berlin für Materialien und Energie GmbH Albert‐Einstein‐Str. 15 12489 Berlin Germany; ^3^ Department of Physics and Astronomy Uppsala University Box 516 75120 Uppsala Sweden; ^4^ Present address: Institut für Methoden und Instrumentierung der Forschung mit Synchrotronstrahlung Helmholtz‐Zentrum Berlin für Materialien und Energie GmbH Albert‐Einstein‐Str. 15 12489 Berlin Germany; ^5^ Present address: Department of Physics and Astronomy Uppsala University Uppsala 75120 Sweden

**Keywords:** catalysis, dichalcogenides, hydrogen evolution reaction, phase transitions, photoelectron spectroscopy

## Abstract

Visible light is shown to create a transient metallic S–Mo–S surface layer on bulk semiconducting p‐doped indirect‐bandgap 2H‐MoS_2_. Optically created electron–hole pairs separate in the surface band bending region of the p‐doped semiconducting crystal causing a transient accumulation of electrons in the surface region. This triggers a reversible 2H‐semiconductor to 1T‐metal phase‐transition of the surface layer. Electron–phonon coupling of the indirect‐bandgap p‐doped 2H‐MoS_2_ enables this efficient pathway even at a low density of excited electrons with a distinct optical excitation threshold and saturation behavior. This mechanism needs to be taken into consideration when describing the surface properties of illuminated p‐doped 2H‐MoS_2_. In particular, light‐induced increased charge mobility and surface activation can cause and enhance the photocatalytic and photoassisted electrochemical hydrogen evolution reaction of water on 2H‐MoS_2_. Generally, it opens up for a way to control not only the surface of p‐doped 2H‐MoS_2_ but also related dichalcogenides and layered systems. The findings are based on the sensitivity of time‐resolved electron spectroscopy for chemical analysis with photon‐energy‐tuneable synchrotron radiation.

## Graphical abstract

The surface S–Mo–S sheet of the p‐doped indirect semiconductor 2H‐MoS_2_ is found to undergo a transient 1T‐MoS_2_‐metallization of the surface layer by optical excitation with distinct threshold and saturation behaviour. This finding builds on the essential sensitivity of time‐resolved electron spectroscopy for chemical analysis with tuneable synchrotron radiation.

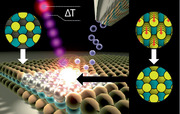

Dichalcogenides MX_2_ (transition metal M and chalcogen X) as van der Waals coupled, layered, quasi 2D materials allow for tailored electronic properties and are thus of high relevance for devices, gas sensors, and chemical processes.^[^ ^1^ ^]^ Underlying is the existence of multiple phases and stacking orders and the ability to be doped and to intercalate as a host material.^[^ ^2^ ^]^ The dichalcogenide molybdenite (MoS_2_) occurs as a thermodynamically stable bulk crystal with an indirect bandgap of 1.2 to 1.3 eV.^[^ ^3^, ^4^, ^5^ ^]^ Its crystal structure consists of stacked S–Mo–S sheets with a trigonal prismatic symmetry of A–B–A stacking, where the sulfur atoms in the top and bottom S‐planes occupy equivalent vertical positions.^[^ ^3^ ^]^ The S–Mo–S sheets have 6.5 Å distance with respect to each other.^[^ ^6^ ^]^ Going from indirect‐bandgap bulk 2H‐MoS_2_ toward a single layer, the bandgap gradually widens, reaching for monolayer MoS_2_ a direct bandgap of 1.9 eV.^[^ ^5^ ^]^ The semiconducting 2H‐MoS_2_ phase supports both n‐ and p‐type doping induced by chemical and physical means.^[^ ^7^, ^8^, ^9^, ^10^, ^11^ ^]^ Intercalation, electronic, optical, and thermal excitations as well as mechanical strain and layer orientation have been reported.^[^ ^3^, ^12^, ^13^, ^14^, ^15^, ^16^ ^]^ Sliding the sulfur atoms of one of the S‐planes in the S–Mo–S layers by 1.82 Å leads to A‐B‐C stacking within the monolayer, where the sulfur atoms occupy the centers of the hexagons of the 2H phase, which results in the metallic 1T‐MoS_2_ phase.^[^ ^3^, ^17^ ^]^ The metallic 1T‐MoS_2_ phase is stabilized by electron injection, for example, by direct electron injection with an electron microscope or electron donation from adsorbed lithium atoms.^[^ ^12^, ^17^, ^18^, ^19^, ^20^, ^21^ ^]^

In the family of the dichalcogenides, MoS_2_ shows the remarkable property of intrinsically driving the photocatalytic and electrochemical hydrogen evolution reaction (HER) of water.^[^ ^22^, ^23^, ^24^, ^25^ ^]^ For the semiconducting 2H‐MoS_2_ phase, sulfided Mo‐edges of the S–Mo–S planes have been identified as active sites^[^ ^25^, ^26^ ^]^ with passive basal planes.^[^ ^6^ ^]^ Charge mobility is one limiting factor to the HER in the naturally occurring semiconducting 2H‐MoS_2_ phase. Thus, one approach to increase the photocatalytic and photoassisted electrochemical HER and overall water splitting rate has been to increase the charge mobility and the number of available active sites.

Indeed, thin‐film growth on a conductive support increases the HER rate.^[^ ^27^, ^28^ ^]^ Preparing the metallic 1T‐MoS_2_ phase (maintaining the number of sulfided Mo‐edge sites) boosts the HER rate further.^[^ ^29^, ^30^, ^31^ ^]^ In addition, the metallic 1T‐MoS_2_ phase even has active basal planes catalyzing the HER.^[^ ^6^, ^29^ ^]^ Moving from single crystal to amorphous MoS_2_ can also increase the HER activity.^[^ ^22^ ^]^ The same applies to the introduction and deposition of well‐coupled transition metal nanostructures (i.e., Au, Ag, Ni, Pd, Pt, Ru, Au glued Ni nanoparticles), combining active sites with sufficient electron mobility.^[^ ^32^, ^33^, ^34^, ^35^, ^36^ ^]^.

In this work, we establish how visible light itself can create a transient metallic top layer on bulk crystalline p‐doped 2H‐MoS_2_. Electron–hole pairs created by optical excitation separate in the surface band bending region of p‐doped semiconducting 2H‐MoS_2_. This causes a transient accumulation of electrons in the surface region, driving the top‐layer within several picoseconds from the p‐doped semiconducting 2H‐MoS_2_ into a sheet of metallic 1T‐MoS_2_ at a remarkably low optical fluence threshold. This mechanism has significant implications on how optically illuminated MoS_2_ surfaces behave.

Essential for this insight has been the selectivity of time‐resolved optically pumped X‐ray photoelectron spectroscopy, which thus far has only been used to study the optically induced valence band dynamics of dichalcogenides.^[^ ^37^, ^38^, ^39^, ^40^ ^]^ Using now core‐level photoelectron spectroscopy yields crucial insight by giving complementary information in three ways: i) The chemical shift of electron spectroscopy for chemical analysis (ESCA) distinguishes through core level binding energies the semiconducting and metallic phases of 2H‐MoS_2_ and 1T‐MoS_2_, respectively. ii) The surface photovoltage shift (SPVS) distinguishes n‐ and p‐doped semiconducting 2H‐MoS_2_ as well as flat‐band semiconducting 2H‐MoS_2_. In particular, it traces excess charge carriers and Fermi level pinning in the semiconducting phases. iii) The universal curve of electron mean free path allows to vary and also match the probing depth of 4 Å by tuning the incidence photon energy. Thereby, one can zoom into the top S–Mo–S sheet, since bulk MoS_2_ has a layer spacing of 6.5 Å between the S–Mo–S sheets.

The temporal evolution of 2H‐MoS_2_ following optical excitation consists of two processes with different time scales: the surface photovoltage shift (SPVS), on characteristic ten‐to‐hundred‐picoseconds (–150 ps up to –10 ps), and a sudden decrease of the binding energy which occurs on the picosecond timescale (–5 ps to 5 ps) as seen by core level electron spectroscopy for chemical analysis with femtosecond X‐ray pulses (**Figure** **1**a–c). We used 5 ps steps close to the temporal overlap and 10 ps steps in the range from –50 ps to –10 ps. Additional delay points have been added at the end of the delay range and close to the temporal overlap. As the femtosecond measurement only detects picosecond dynamics, we switch to more intense picosecond X‐ray pulses and delay steps of 10 ps (Figure 1d,e). The same temporal evolution of binding energy as in Figure 1b,c is observed accompanied by additional spectral peak broadening due to the 50 ps X‐ray pulses themselves.

**Figure 1:**
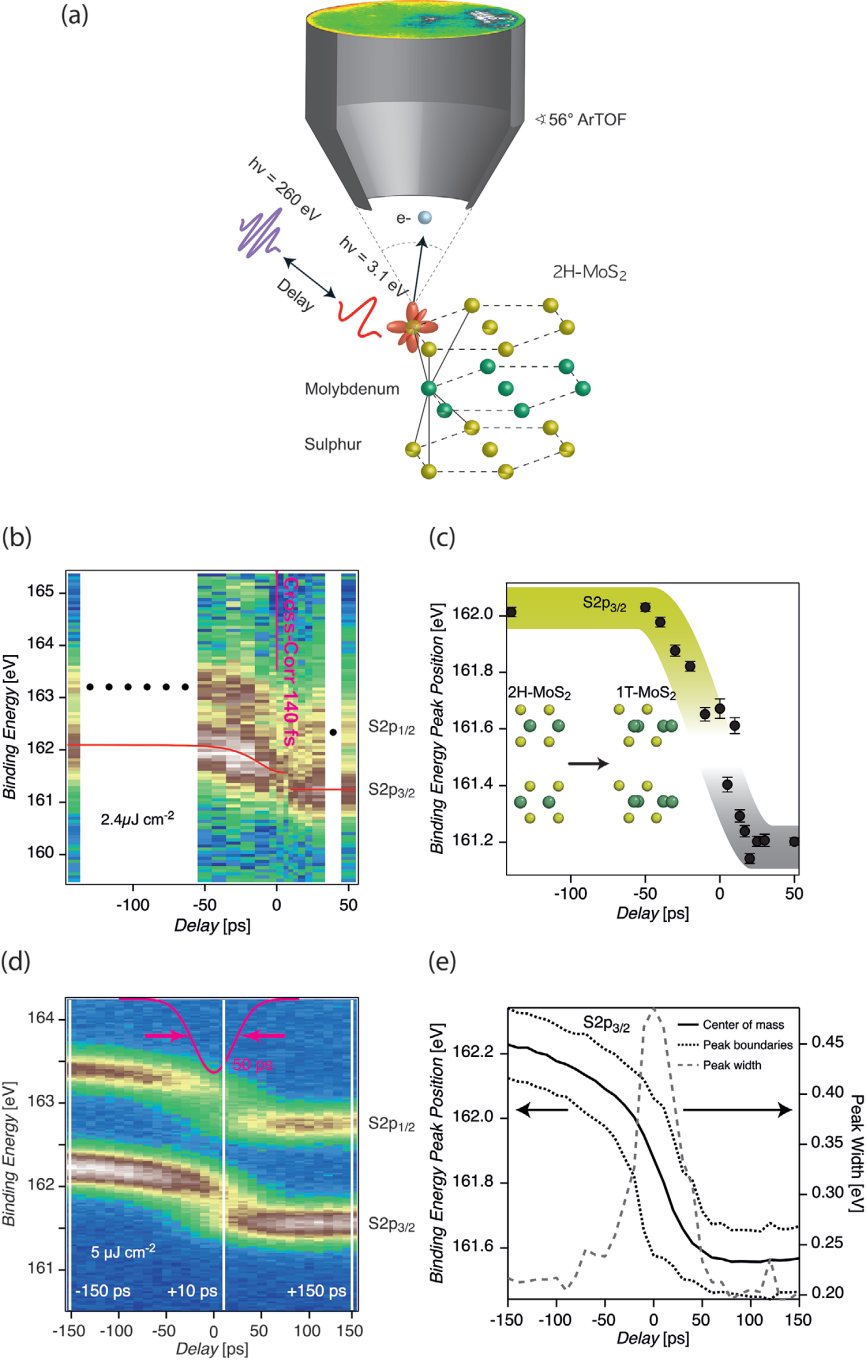
2H‐semiconductor to 1T‐metal top layer phase transition in p‐doped bulk 2H‐MoS_2_ from time‐resolved ESCA: a) The principle of time‐resolved ESCA: optically pumped bulk p‐doped 2H‐MoS_2_ probed by X‐ray photoelectron spectroscopy at the S2p core levels with an angle‐resolved time‐of‐flight (ArTOF) electron spectrometer. b) Femtosecond time resolved S2p core level lines at 140 fs X‐ray/optical cross‐correlation (red line guide to the eye of experimental peak position). c) Temporal evolution of S2p_3/2_ peak positions (as resulting from a single‐component Gaussian fit) using the spectra from (b) shows the surface photovoltage shift and a fast phase transition. d) Picosecond time‐resolved S2p core‐level lines at 50 ps X‐ray/optical cross‐correlation. The S2p multiplet shifts to lower binding energy when approaching the temporal overlap. e) Center of mass (CoM) position of the S2p_3/2_ core level and width. The large increase in the peak width is revelatory of the 2H to 1T phase transition; the CoM peak position reflects the dynamics of both, the phase transition and the SPVS.

Analyzing the dynamics now by fitting a pair of spin‐orbit split Voigt peaks to the spectral data allows us to decompose the transient region with the distinct broadening into different species of sulfur atoms. This is the strength of ESCA since the core levels are sensitive to the different chemical surroundings of the investigated sulfur species. To illustrate the method, we chose the distinct delays marked in Figure 1d with the white lines and which are shown in **Figure** **2**a,c,e in detail. For the negative delay of –150 ps, panel (a) shows the S2*p* multiplet of semiconducting p‐doped bulk 2H‐MoS_2_ (yellow‐green).^[^ ^19^ ^]^ At large positive delay of +150 ps after optical excitation (panel e), the peaks are found at about 740 meV lower binding energy (gray). For +10 ps pump–probe delay, we observe two components: a fraction appearing at about 740 meV lower binding energy and the optically pumped semiconducting p‐doped 2H‐MoS_2_ fraction, shifted by 300 meV to lower binding energy in comparison to unpumped case. Both fractions have equal intensities at this delay point.

**Figure 2:**
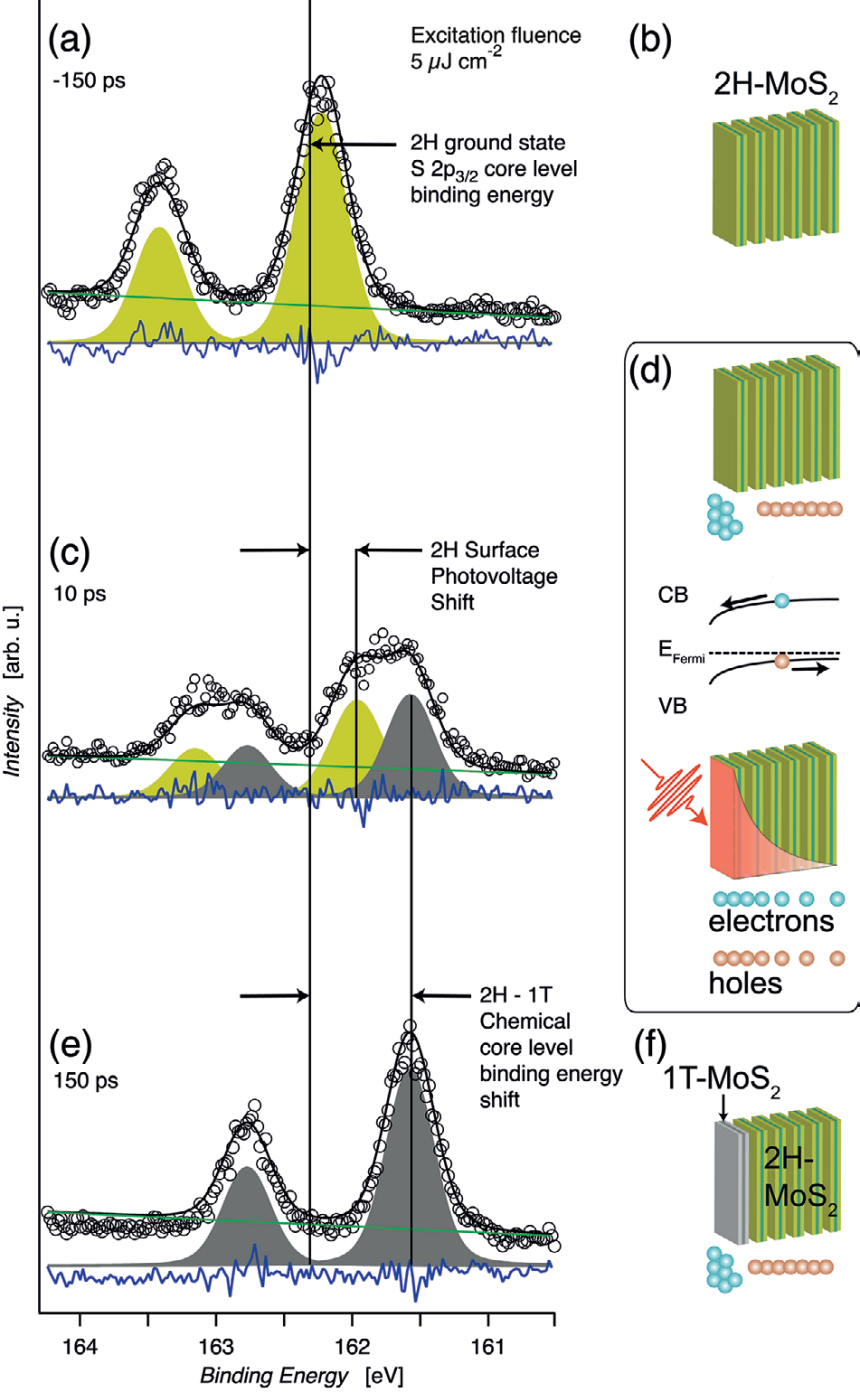
Electron accumulation in the surface layer as the driver of the top‐layer 2H‐semiconductor to 1T‐metal phase transition in p‐doped bulk MoS_2_: a) S2p_3/2_ and S2p_1/2_ core level spectrum of p‐doped MoS_2_ in the semiconducting 2H ground state. b) Sketch of S–Mo–S layers in the 2H ground state. c) Laser excitation leads to surface photovoltage shift (SPVS) of p‐doped semiconducting 2H‐MoS_2_ (marked yellow‐green) and occurrence of chemically shifted metallic 1T‐MoS_2_ S2p_3/2_ and S2p_1/2_ core level lines (marked gray). d) Creation of electron–hole pairs with an exponential excitation profile from optical penetration depth; separation in the surface region of p‐doped 2H‐MoS_2_: electrons in the conduction band (CB) accumulate at surface, holes in the valence band (VB) are pushed into bulk. Surface electron accumulation leads to a 0.14 eV SPVS in S2p_3/2_ and S2p_1/2_ core level binding energies of the optically excited semiconducting 2H phase; surface electron accumulation eventually generates and stabilizes the 1T metallic phase. e,f) 150 ps after the excitation; the peaks arising from the 2H phase have completely disappeared indicating that the surface layer is now fully in the metallic 1T phase.

These transient shifts in binding energies are observed to be highly fluence‐dependent. **Figure** **3**a shows how the S2p_3/2_ peak position decreases linearly in binding energy due to the SPVS in the p‐doped 2H‐MoS_2_ for fluences up to 2.6 μJ cm^−2^. The shift is on the order of 310 meV at this fluence. Increasing the fluence above 2.7 μJ cm^−2^ leads to a shift of about 680 meV deviating from the linear relationship observed at lower fluences. The binding energy shift reaches a saturation value of 830 meV above 4.2 μJ cm^−2^. Further increase in excitation fluence does not further alter the S2p_3/2_ core level binding energy.

**Figure 3:**
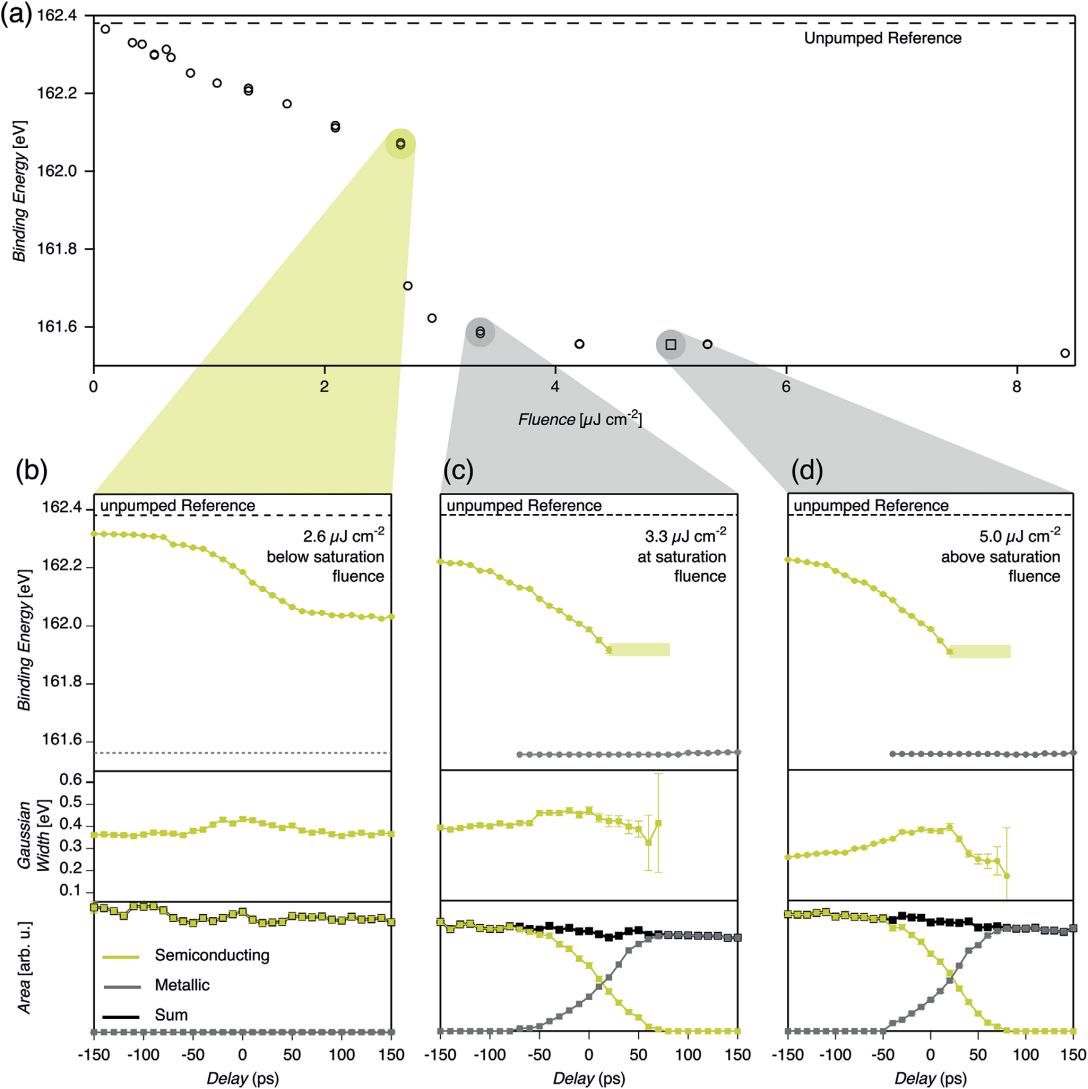
Excitation fluence dependence, threshold, and saturation of top‐layer 2H‐semiconductor to 1T‐metal phase transition in p‐doped bulk MoS_2_: a) S2p_3/2_ core level binding energy at +150 ps delay as a function of optical excitation fluence. The top‐layer 2H‐semiconductor to 1T‐metal phase transition threshold is at 2.6 μJ cm^−2^ and saturation is reached above 3.3 μJ cm^−2^. Since every absorbed optical photon leads to the creation of an electron–hole pair, we can give an estimate of the surface electron density. The intensity threshold of 2.6 μJ cm^−2^ corresponds to 5.2 × 10^12^ photons cm^−2^. Considering a reflectivity of the sample of about 50%, this leads to an upper limit of about 2.6 × 10^12^ photons cm^−2^ being absorbed. This sets the upper limit of laser‐induced surface electron doping density assuming complete accumulation of electrons at the very surface. b) Below the saturation fluence threshold (2.6 μJ cm^−2^), p‐doped MoS_2_ stays in the semiconducting 2H phase for all delays as seen from S2p_3/2_ core level binding energies, Gaussian peak width contribution, and intensities. c) At saturation fluence threshold (3.3 μJ cm^−2^) and d) above (5 μJ cm^−2^) the top‐layer is fully converted into the metallic 1T phase.

The temporal evolution of the S2p_3/2_ core level binding energy on the picosecond timescale is displayed in Figure 3b–d. The ensuing peak intensities representing the p‐doped semiconducting 2H‐MoS_2_ and the fraction appear at a distinct lower binding energy. Staying below the optical excitation threshold at 2.6 μJ cm^−2^, only S2p_3/2_ core level binding energies related to p‐doped semiconducting 2H‐MoS_2_ with the typical SPVS behavior can be observed. No intensity of the species appearing at lower binding energy can be detected (Figure 3b). At 3.3 μJ cm^2^ optical excitation fluence we observe, as a function of temporal delay, progressive disappearance of the S2p_3/2_ peak associated to p‐doped semiconducting 2H‐MoS_2_ and the concomitant appearance of the peak with a lower binding energy. In fact, this behavior stays very similar for all optical excitation fluences above 3.3 μJ cm^−2^. Comparing the delay‐dependent evolution of the binding energies for the three fluences, we note that the shift of the semiconducting phase observed below the threshold (b) is slightly lower than above this threshold. In contrast, above the threshold (c) and in deep saturation (d), the SPVS are almost equal due to a flat band condition. The fraction with the lower binding energy appears at about the same binding energy for both cases (c,d) well separated from the value of the 2H fraction. Furthermore, we observe a delay and fluence‐dependent change in the width of the peaks associated with the semiconducting phase. Namely, there is a slight increase of the Gaussian component of the Voigt fit in the vicinity of the temporal overlap between the two pulses. This can be attributed to the averaging of the SPVS due to the X‐ray pulse length of 50 ps.

The relaxation back to the ground state can be observed in **Figure** **4**. It is important to emphasize the distinctly different behavior for the relaxation at low optical excitation fluences staying within the p‐doped semiconductor MoS_2_ phase (yellow‐green shaded area) and at optical excitation fluences above the onset of the saturation regime (gray shaded area). First of all, we observe that the process is fully reversible in both regimes. All traces are separated in binding energy for all delays in the low excitation regime. Especially, the binding energies at a delay of 800 ns do not overlap. The decay dynamics completely change in the high fluence regime. Although the binding energies can still be different for a delay of 150 ps due to the saturation effect, the binding energies collapse onto the same value at a delay of 800 ns. Furthermore, for the two highest fluences shown here, both traces seem to collapse already at a delay of 12 ns. For delays larger than 800 ns, the three shown traces spanning the saturation regime starting at the onset of the sudden change in binding energy have very similar binding energies for all delays up to 166 μs.

**Figure 4:**
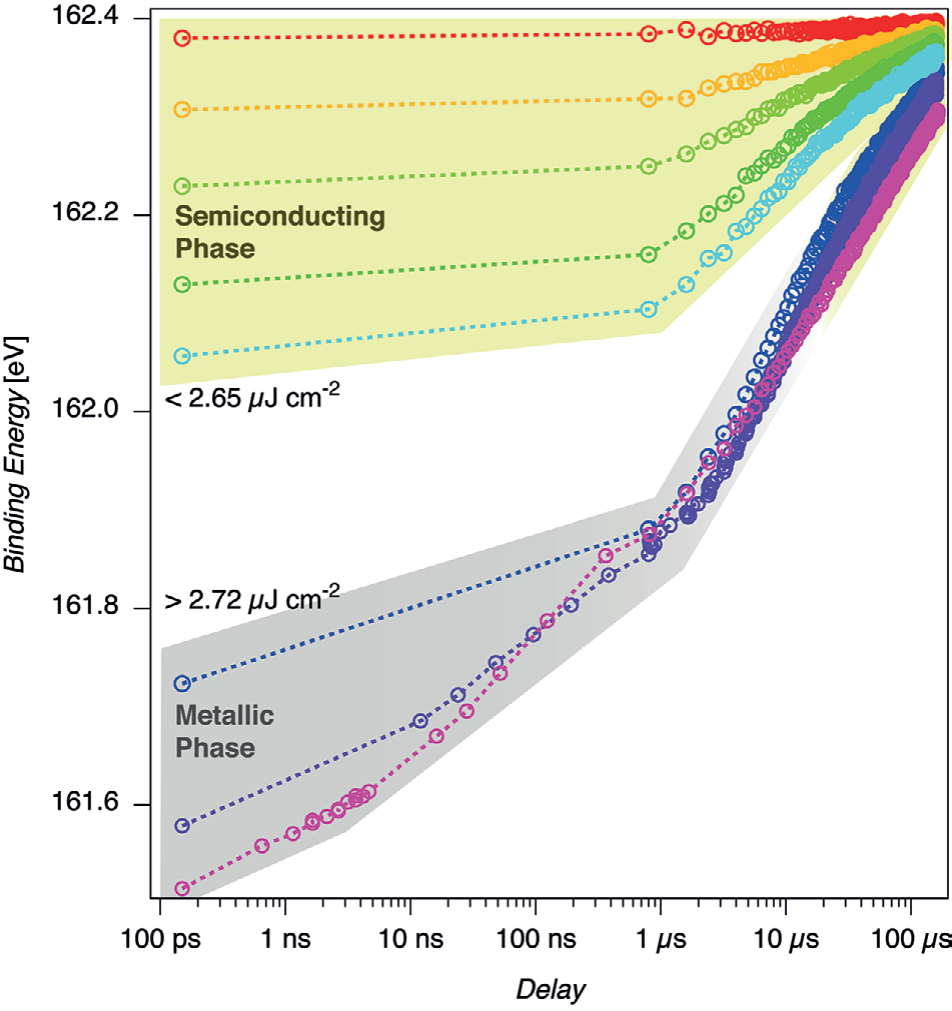
Reequilibration to the 2H semiconducting ground state from different excited phases as a function of fluence: a) S2p_3/2_ core level binding energies spanning up to 166 μs optical‐pump/X‐ray‐probe delay with a spacing of 800 ns. These points are accumulated at the repetition rate of the laser, that is, 6 kHz. Additional delay points have been added for the two highest fluences. The uppermost group of data (yellow‐green shaded area) show how the semiconducting phase relaxes during the time span between two consecutive laser pulses. The lower points (gray shaded area) refer to the relaxation of the metallic phase. For fluences below 2.65 μJ cm^−2^, the change in binding energy within the first 800 ns stays below 50 meV. For fluences above 2.72 μJ cm^−2^, the binding energy change within the first 800 ns exceeds 150 meV, reaching in the saturation regime up to 360 meV. Moreover, whereas each of the decay curves of the semiconducting phase are well separated, the decay curves for the metallic phase seem to merge after about 12 ns to the same intermediate state which further decays toward the unexcited phase. The different behavior of the decay in the two regimes might indicate coupling between the S–Mo–S top‐layer and lower‐laying layers, where eventually at fluences exceeding the saturation fluence threshold also deeper layers undergo the same 2H to 1T phase transition.

In **Figure** **5**a, we show the binding energy of the S2p_3/2_ core level as a function of laser fluence at the positive delays of 150 ps (empty, orange circles) and of 800 ns (filled, blue squares) as indicated in the inset. The binding energy in the high fluence regime varies only little with fluence, but with delay, it varies significantly. The binding energy at a delay of 800 ns reaches its saturation almost immediately above the threshold fluence. However, for the short delay of 150 ps, the saturation of the binding energy is reached at a higher fluence of about 4 μJ cm^−2^. In Figure 5b, we show the correlation between the binding energies at the two different delay times. We observe a linear behavior in both, the low fluence and the high fluence regime. However, the slopes in the two regimes are significantly different as indicated by the linear regression fits to the data points.

**Figure 5:**
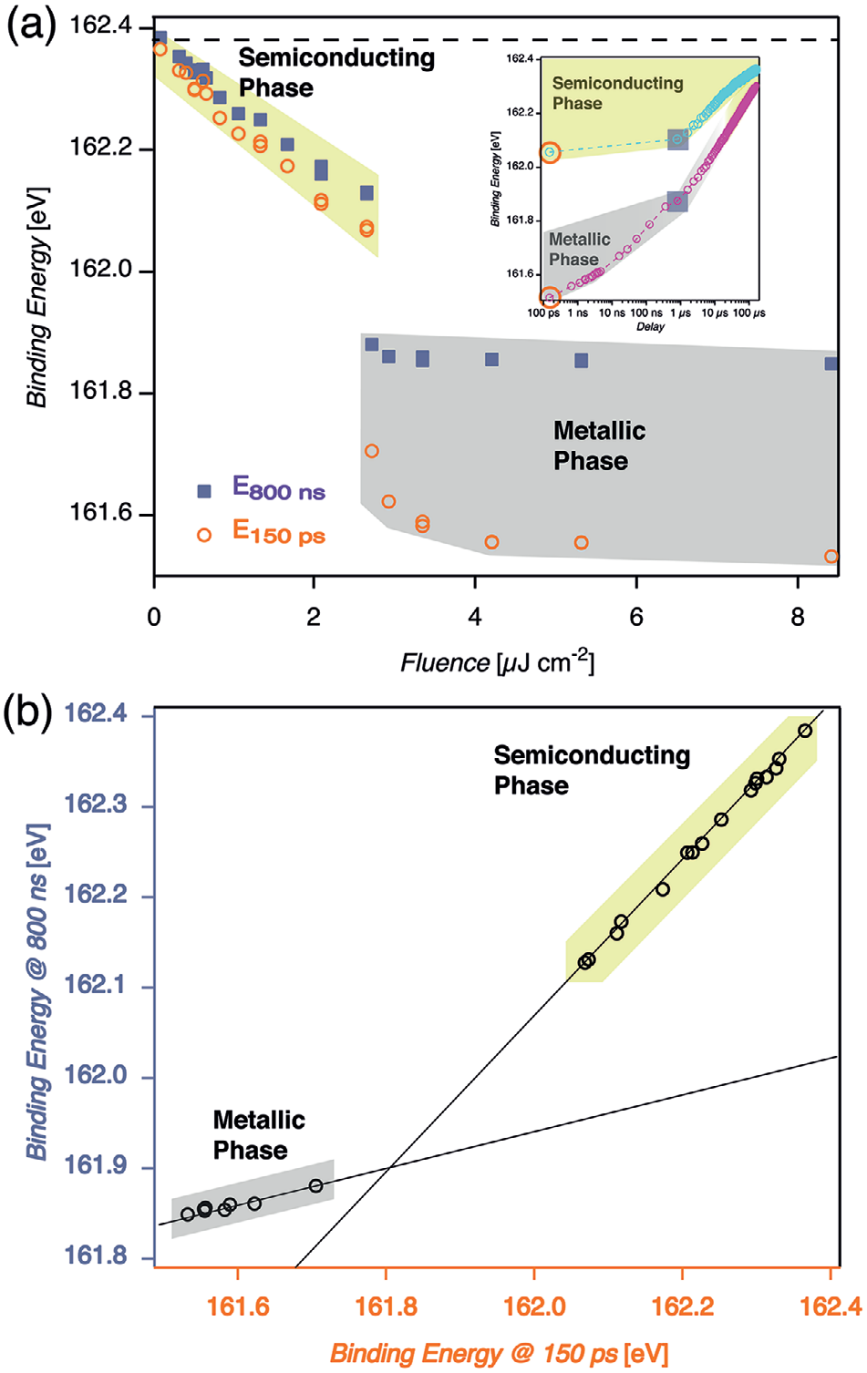
Correlations during reequilibration of the semiconducting and top‐layer metallic excited phases back to the p‐doped semiconductor MoS_2_ ground state: a) overview indicating the regions of prompt response to optical excitation 150 ps (orange, opened circles) and dampened equilibration 800 ns (blue, filled squares) as a function of fluence. The insert is a simplified version of Figure 4 for illustration. b) Correlation between the prompt (150 ps) and dampened (800 ns) semiconducting and metallic materials fractions as seen from the prompt and dampened S2p_3/2_ core level binding energies.

The presented experimental data shows that semiconducting p‐doped bulk MoS_2_ has a substantially different behavior upon laser excitation deviating from other well‐studied systems like p‐doped GaAs. A comparison to p‐doped GaAs is given in the supplement. For low fluences, the SPVS in p‐doped MoS_2_ shows a linear fluence dependence as expected for a p‐doped semiconductor. However, increasing the fluence further leads to a sudden reduction of the binding energy. The saturation binding energy in this regime is similar to the binding energy of the metallic 1T phase of MoS_2_.^[^ ^19^ ^]^ Moreover, investigating the dynamics of the process as a function of pump–probe delay and pump fluence shows that the core‐level of the 2H phase does not gradually shift to the lower binding energy position for the fluences above the threshold value. Instead, the data acquired using the femtosecond X‐ray pulses indicate a sudden change of the binding energy within a few picoseconds. The data using the picosecond X‐ray pulses further strengthen this observation since both species coexist at the temporal overlap and are distinguishable in the data. Finally, the dynamics of the decay changed dramatically exceeding the threshold value. For low fluences, the delay traces are well separated for all fluences and delay times while above the threshold the traces merge at larger delays. The substantial change of the dynamics of the decay is further strengthened by plotting the binding energy at a delay of 800 ns as a function of the binding energy at a delay of 150 ps. Here, we observe that both, the low and the high fluence regime fall into two well separated groups. Additionally, both groups have a linear dependence, but with very different slopes. These observations using time‐resolved ESCA suggest that we observe a phase transition of 2H‐MoS_2_ to 1T‐MoS_2_ in the top layer of our sample.

The underlying physical mechanism is schematically depicted in Figure 2b,d,f. In the semiconducting p‐doped bulk 2H‐MoS_2_ sample (Figure 2b), absorption of optical radiation across the bandgap leads to electron (marked blue)–hole (marked red) pair formation following the exponential depth profile of the absorbed optical radiation (red) (Figure 2d).

As a result of the downward band bending of p‐doped 2H‐MoS_2_, the electrons in the conduction band are pulled toward the surface layer, whereas the holes in the valence band are pushed into the bulk (Figure 2d) under optical excitation. This relaxes the initial band bending in the surface region leading to a relative negative binding energy shift of the S2p core levels of 2H‐MoS_2_ with respect to the unpumped case. The direction of the SPVS reflects the p‐type nature of the semiconductor following the established mechanism of SPVS on intrinsically doped semiconductors.^[^ ^41^ ^]^ Moreover, due to the indirect bandgap in bulk MoS_2_, the electrons (holes) move in k‐space toward the conduction band (CB) minimum (valence band [VB] maximum). During this process, the electrons and holes excite phonons, which puts the lattice into an excited state thereby mitigating a potential energy barrier. Together with the build‐up of electrons in the top‐layer, this leads to the formation of the metastable, metallic 1T phase of MoS_2_ (Figure 2f). This process parallels the known phase transition from 2H to 1T by direct electron injection from, for example, an electron microscope or electron donation from adsorbed lithium atoms.^[^ ^18^, ^20^, ^42^, ^43^ ^]^ We thus create a transient, controllable, and reversible electron‐induced phase transition by the described ultrafast photodriven electron accumulation pathway within the surface region of p‐doped 2H‐MoS_2_.

We established the transient picosecond top‐layer 2H‐semiconductor to 1T‐metal phase‐transition in p‐doped MoS_2_ by photodriven surface electron accumulation, using femtosecond‐to‐microsecond time‐resolved core‐level electron spectroscopy for chemical analysis. A distinct excitation threshold at low photon fluence relates this mechanism to the reported photoassisted and photocatalytic HER rate enhancement of MoS_2_ when exposed to visible light. First, a gradual light‐induced increase in charge mobility in the optically created 1T top layer can enhance the HER rate at the existing active sites. Second, an enhancement of the HER rate is driven by the fact that in the 1T phase the full basal plane is catalytically active whereas for the 2H phase only edge sites contribute. Generally, the described mechanism of photoinduced top layer electron accumulation and top layer modification in a p‐doped dichalcogenide defines a conceptual framework to enhance a wide range of catalytic and device properties.

## Experimental Section

The experiments were performed at the Plane Grating Monochromator (PGM) branch of the “FemtoSpeX” facility at Bessy II (UE56/1‐PGM)^[^ ^44^ ^]^ using the “FemtoSpeX Molecules and Surfaces” endstation. The monochromator was equipped with three gratings: 1200 lines mm^−1^, 400 lines mm^−1^, and 150 lines mm^−1^. The regular fill pattern of BESSY II consisted of several parts: The multibunch part, with 2 ns spacing between consecutive bunches, an ion clearing gap of 200 ns, and several isolated bunches with a higher current. Two of these isolated bunches were located within the ion clearing gap and one of them was excited by a radio transmitter effectively allowing single bunch operation at the endstation. This method is called pulse picking by resonant excitation (PPRE).^[^ ^45^ ^]^ The remaining isolated bunches were located in the multibunch part and were dedicated to the femtosecond slicing process. The strength of this beamline was thus the availability of regular single pulse synchrotron radiation (50 ps FWHM) and femtosecond X‐ray pulses (≈120 fs FWHM) with variable polarization by tuning the electron beam and beamline optics.^[^ ^44^, ^45^ ^]^ The 150 lines mm^−1^ grating was used for the measurements using the femtosecond X‐ray pulses, yielding a resolution of *E*/Δ*E* ≃ 10^3^ and the 1200 lines mm^−1^ grating for the picosecond measurements. A photon energy of 260 eV and vertical polarization were used. Combining the pulses delivered by the synchrotron, a synchronized femtosecond optical laser available at the beamline and a high transmission angle‐resolved time‐of‐flight electron spectrometer (ArToF) with an acceptance of 56° enabled the investigation of the dynamics of core levels.^[^ ^46^, ^47^, ^48^, ^49^ ^]^ The pump laser system consisted of a Ti:sapphire amplifier (Legend Elite Duo, COHERENT), which was seeded by the same oscillator (Micra, COHERENT, 800 nm) as the laser providing the pulses for the slicing process.^[^ ^44^ ^]^ The oscillator, running at 83.3 MHz ($\hat{=}$12 ns), was phase‐locked to the master clock of the storage ring. The amplifier delivered pulses with a fluence of up to ≈1.8 mJ at a maximum frequency of 6 kHz at a center wavelength of 800 nm (FWHM ≈45 fs). The 2nd harmonic of these pulses (400 nm, FWHM ≈70 fs), generated by using a BBO crystal, was used as a pump. The laser fluence could be adjusted by varying the polarization of the laser beam in front of two thin film polarizers (LAYERTEC, 400nm, 56°, *R*_*s*_ > 99.8%, *R*_p_ < 2%). Additional reflective UV grade ND filters were used to further reduce the fluence (Thorlabs). The fluence was measured before the incoupling in the beamline (Gentec UP19K‐15S‐W5‐D0 and XLP12‐3S‐H2‐D0). The fluence after the ND filter was calculated using the fluence without the filters and the appropriate attenuation coefficient. The laser pulses and the synchrotron radiation pulses could be delayed relative to each other using an optical delay stage spanning a delay range of up to 4.4 ns with femtosecond resolution (Newport DL325, MIM 0.5 fs). Since the laser system was running at 6 kHz and the synchrotron pulses arrived at a repetition rate of 1.25 MHz in PPRE mode, the decay of the optically excited system could be efficiently tracked in 800 ns steps up to a timescale of about 200 μs with a single measurement at a fixed optical delay stage. This allowed to compare the dynamics of the decay for different fluences and to extract correlations of binding energies at different delay points. Moreover, the laser timing could be shifted electronically in steps of 12 ns, spanning the delay range from 12 ns up to 800 ns. Finally, the delay range below 12 ns could be reached by shifting the synchronization of the phase lock continuously. The combination of the ≈120 fs long X‐ray pulses and the ≈70 fs long laser pulses led to a temporal resolution of ≈140 fs. Using the PPRE mode, the temporal resolution was dominated by the pulse length of the X‐ray pulses of 50 ps.

Complementary static properties of MoS_2_ were investigated at the LowDose endstation located at the UBjL beamline PM4 at BESSY II. The data acquisition software was tested and developed at the LowDose endstation. The wide‐angle lens tables of the spectrometer were improved in cooperation with the endstation CoESCA located at the beamline UE52‐PGM at BESSY II.

Commercially available slightly p‐doped MoS_2_ bulk samples (2D semiconductors) were used for the experiment. The doping was on the order of 10^15^ to 10^16^ cm^−3^. The samples were annealed at 120 °C in vacuum before cleaving at a base pressure <1 × 10^−9^ mbar. Several samples were investigated and showed the same behavior with slight variations of the laser fluence threshold.

The data set was analyzed using the ArToF Loader and Analysis package for IGOR^[^ ^48^ ^]^. Curve fitting to the individual spectra was done using SPANCF^[^ ^50^ ^]^. The fits to the two S2p core levels were Voigt fits with a linear background. The spin‐orbit splitting was kept as a fixed parameter to be 1.19 eV. The intensity ratio between the spin‐orbit split core‐levels was also fixed to be 1:2. The Voigt line profiles were dominated by the experimental Gaussian peak width contribution of 0.4 eV FWHM over the S2*p* Lorentzian life time broadening of 0.08 eV FWHM.

## Reference Measurement on GaAs

: For reference, p‐doped GaAs (see Supporting Information) were also investigated. The GaA wafers were oriented in the (100) direction with a miscut of 2° in the (111) direction. The dopant was zinc with a concentration of 1–80 × 10^18^ cm^−3^. The etch pit density was measured to be <5 × 10^4^ cm^−2^. Commercially available samples obtained from CrysTec were used. The protective varnish was removed by using an ultrasonic shaker and an organic solvent. The samples were sputtered in vacuum in order to remove the surface contaminants.

## Conflict of Interest

The authors declare no conflict of interest.

## Supporting information

Supporting Information

## Data Availability

Raw data were generated at BESSY II synchrotron radiation facility (beamline UE56/1‐PGM). Derived data supporting the findings of this study are available from the corresponding author upon request.
