# Supplementary material for: Photodriven Transient Picosecond Top‐Layer Semiconductor to Metal Phase‐Transition in p‐Doped Molybdenum Disulfide
Source: Adv Mater. 2021 Mar 4;33(14):2006957. doi: 10.1002/adma.202006957 (PMC11469269; doi:10.1002/adma.202006957)
Supplement: Supplementary file 1 — Supporting Information [file ADMA-33-2006957-s001.pdf]

© 2021 Wiley-VCH GmbH

ADVANCED MATERIALS

Supporting Information

for *Adv. Mater.,* DOI: 10.1002/adma.202006957

Photodriven Transient Picosecond Top-Layer Semiconductor to Metal Phase-Transition in p-Doped Molybdenum Disulfide

Nomi L. A. N. Sorgenfrei, Erika Giangrisostomi, Raphael M. Jay, Danilo Kühn, Stefan Neppl, Ruslan Ovsyannikov, Hikmet Sezen, Svante Svensson, and Alexander Föhlisch*

Photodriven Transient Picosecond Top-Layer Semiconductor to Metal Phase-Transition in p-Doped Molybdenum Disulfide

Nomi L. A. N. Sorgenfrei Erika Giangrisostomi Raphael M. Jay Danilo Kühn Stefan Neppl Ruslan Ovsyannikov Hikmet Sezen Svante Svensson Alexander Föhlisch*

Dr. N. Sorgenfrei
Institut für Physik und Astronomie
Universität Potsdam, Karl-Liebknecht-Straße 24/25
14476 Potsdam
Germany
Current address: Institut für Methoden und Instrumentierung der Forschung mit Synchrotronstrahlung
Helmholtz-Zentrum Berlin für Materialien und Energie GmbH
Albert-Einstein-Str. 15
12489 Berlin
Germany
Email Address: nomi.sorgenfrei@helmholtz-berlin.de

Dr. E. Giangrisostomi, Dr. S. Neppl, Dr. R. Ovsyannikov, Dr. H. Sezen
Institut für Methoden und Instrumentierung der Forschung mit Synchrotronstrahlung
Helmholtz-Zentrum Berlin für Materialien und Energie GmbH
Albert-Einstein-Str. 15
12489 Berlin
Germany

Dr. R. Jay
Institut für Methoden und Instrumentierung der Forschung mit Synchrotronstrahlung
Helmholtz-Zentrum Berlin für Materialien und Energie GmbH
Albert-Einstein-Str. 15
12489 Berlin
Germany
Current address: Department of Physics and Astronomy
Uppsala University
75120 Uppsala
Sweden

Dr. D. Kühn
Institut für Physik und Astronomie
Universität Potsdam, Karl-Liebknecht-Straße 24/25
14476 Potsdam
Germany

Prof. Dr. S. Svensson
Department of Physics and Astronomy
Uppsala University
Box 516
75120 Uppsala
Sweden Institut für Methoden und Instrumentierung der Forschung mit Synchrotronstrahlung
Helmholtz-Zentrum Berlin für Materialien und Energie GmbH
Albert-Einstein-Str. 15
12489 Berlin
Germany

Prof. Dr. A. Föhlisch
Institut für Physik und Astronomie
Universität Potsdam, Karl-Liebknecht-Straße 24/25
14476 Potsdam
Germany
Institut für Methoden und Instrumentierung der Forschung mit Synchrotronstrahlung
Helmholtz-Zentrum Berlin für Materialien und Energie GmbH
Albert-Einstein-Str. 15
12489 Berlin
Germany

1 Depletion layer thickness of the MoS_2 samples

Our samples were delivered with a small p-doping. This has to be considered in order to understand the results, since this p-doping affects the behaviour of the semiconducting phase and it is also expected to change after treatment of the samples i.e. after annealing. The intrinsic density of charges of a semiconductor is given by:

$$n_i = N_s \cdot exp\left\{ -\frac{E_g}{2k_bT} \right\} \tag{1}$$

with N_s being the effectively available states, E_g is the band gap, k_b the Boltzmann constant and T the temperature.

$$N_S = 2 \cdot \left\{ \frac{m^* k_b T}{2\pi\hbar^2} \right\}^{\frac{3}{2}} \tag{2}$$

The band gap in MoS_2 is 1.2eV, the effective mass of the holes (electrons) is $0.56m_0$ $(0.55m_0)$[7]. Using equations 1 and 2 this results in a intrinsic density of charges of $n_i = 875 \cdot 10^6 cm^{-3}$.
The separation of the Fermi level from the intrinsic Fermi level is given by:

$$(E_F - E_i) = k_bT \ln\left(\frac{n_0}{n_i}\right) = 0.026eV \cdot \ln\left(\frac{n_0}{n_i}\right) \tag{3}$$

where n_0 is the doping density.
According to the manufacturer our samples are p-doped in the range from $1 \cdot 10^{15} cm^{-3}$ up to $1 \cdot 10^{16} cm^{-3}$. This gives a shift of the Fermi level of about 360meV up to 420meV.
The depletion layer thickness is given by

$$d = \sqrt{\frac{2V\epsilon\epsilon_0}{e_0 n_0}} \tag{4}$$

with V being the shift of the bands at the surface.
Using the numbers for the doping and the observed shift of about 300meV below the phase transition threshold, and a dielectric constant of 3.5[5], the depletion layer thickness varies between 110nm and 340nm. Using the appropriate shift of the Fermi level, the depletion layer thickness varies between 130nm and 375nm. Using other values for the dielectric constant from the literature[6;7] the thickness varies between 110nm and 310nm.
The absorption coefficient of 400nm light in MoS_2 is about $1.0 \cdot 10^6 cm^{-1}$[8]. Using the Beer-Lambert law it is evident that the non-reflected part of the impinging fluence is fully absorped within the depletion layer.

2 Decay

As already mentioned in the main text, the decay process changes dramatically exceeding the pump threshold value of about $2.7\mu J/cm^2$. Here, we provide further illustration of this process by investigating correlations of binding energies at different delay times. Namely, we consider the binding energy difference

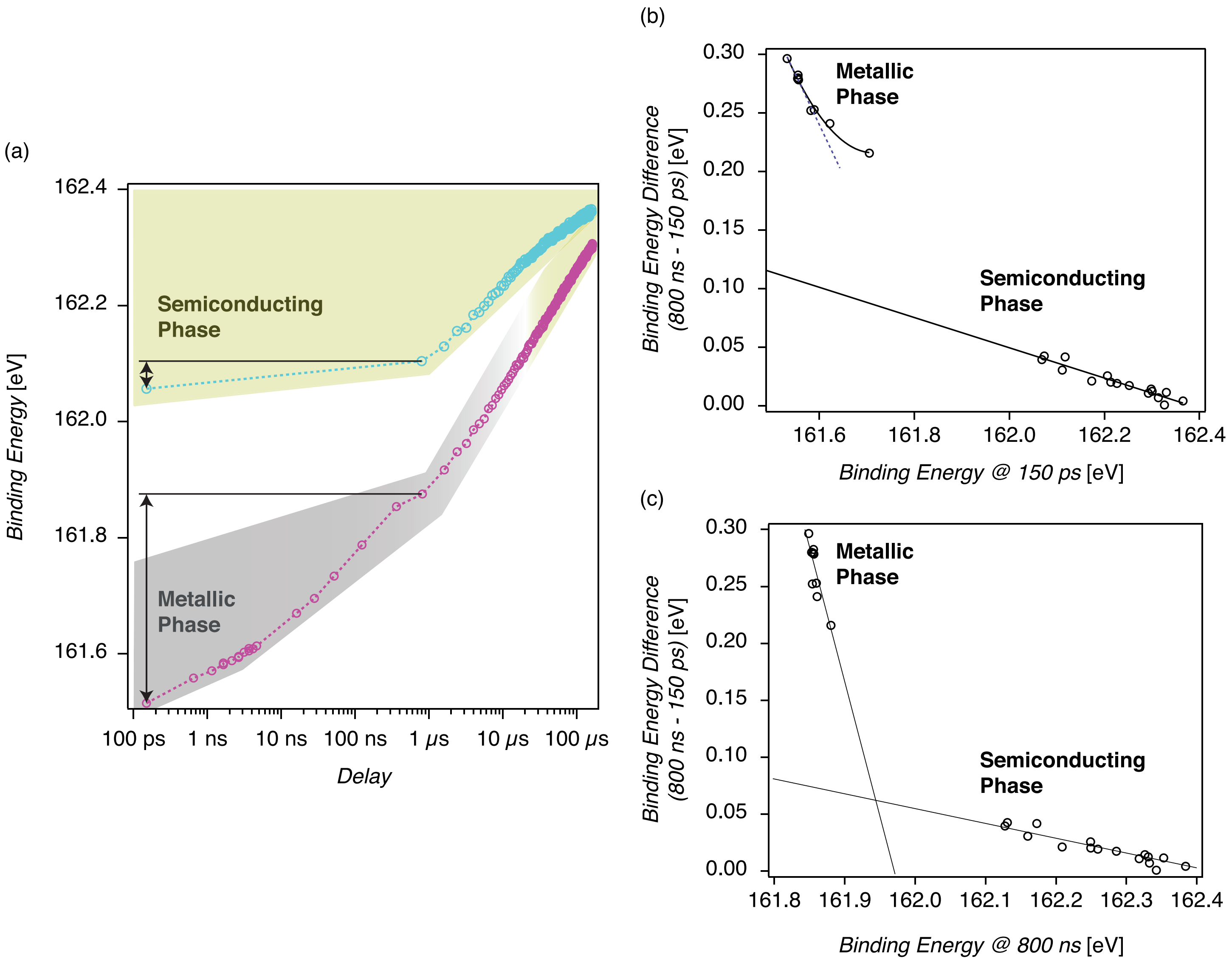

Figure 1: **Differential Decay Analysis:** In panel (a) the difference which is plotted versus binding energies at different delay times is illustrated. Panel (b) shows the relationship between this difference and the initial binding energy directly after the pump pulse. In panel (c) we illustrate how this behavior changes when plotting the difference versus the binding energy after partial relaxation.

between +150ps and +800ns. In Fig. 1b) we show how this difference evolves as a function of the binding energy directly after the pump pulse. Below the threshold, the absolute value of the difference increases linearly with decreasing binding energy (i.e. increasing pump fluence). This can be understood by the fact that by increasing the pump fluence we create more electron-hole pairs which decrease the band bending. However, above the threshold the same relationship seems to be nonlinear (blue dashed line vs black solid curve, which is a quadratic fit). In Fig. 1c) we show the same binding energy difference plotted versus the binding energy at $+800ns$. Again, for fluences below the threshold the dependency is linear. However, for fluences above the threshold, we now observe also a linear behavior, but with a completely different slope. These findings indicate that by exceeding the threshold value the decay process changes dramatically.

3 Time-resolved measurements on p-doped bulk GaAs

The measurements of the SPVS on p-doped GaAs have been performed in low-α mode of BESSY II with a bunch length of $\sim 20ps$ (FWHM). The maximum achievable shift observed for this sample was of about

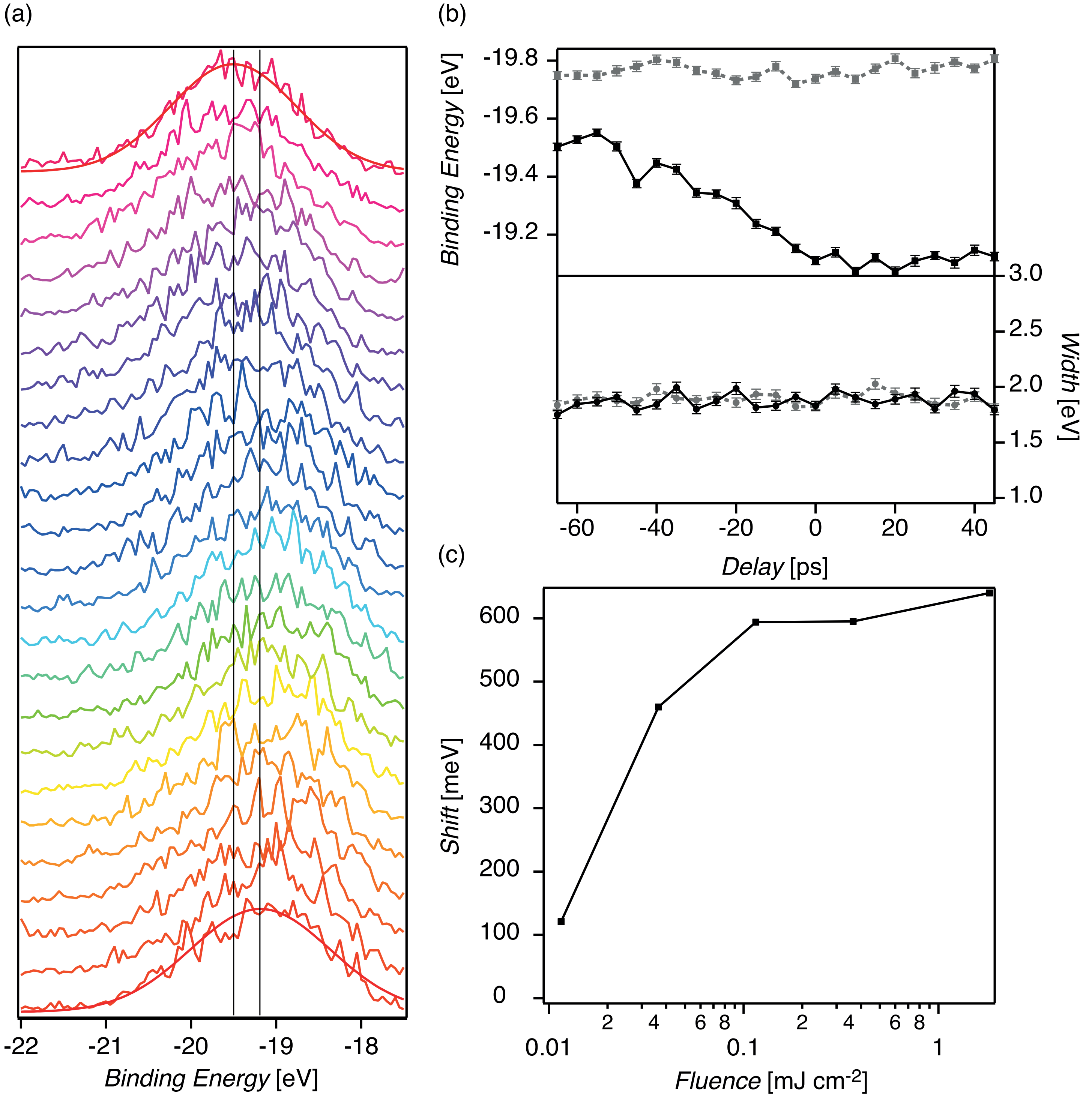

Figure 2: **SPVS in a bulk semiconductor:** Panel (a) shows the individual spectra of the Ga3d region for different delay times (negative delay at the bottom). The black lines illustrate the shift between the unpumped and the pumped spectra. Panel (b) illustrates the shift and the broadening as a function of delay for the pumped (black solid line) and the unpumped (grey dashed line) case. Panel (c) depicts the fluence dependency which clearly shows the expected logarithmic behavior.

$0.6eV$, as it can be seen in Fig. 2c), and was reached at a fluence of about $100\mu J/cm^2$. This fluence is about a factor of 40 bigger than the threshold fluence we observed for MoS_2. Moreover, the maximum shift we observed in MoS_2 was about $0.9eV$. Fig. 2a) shows the different spectra of a delay scan. In Fig. 2b) we show the position (top) and broadening (bottom) for the "pumped" x-ray revolutions (black solid line) and the "unpumped" x-ray revolutions about $166\mu s$ later (grey dashed line). The fit shows no broadening within the error bars. Even though the spectrum of the Ga3d is much broader than the S2p core

levels of MoS_2 we can use this as a reference sample. An alternative explanation for the broadening in MoS_2 would be a smearing due to the pulse length. This type of broadening would be well visible even with the broad Ga3*d* spectrum. In Fig. 2c we show the fluence dependency of the binding energy shift as a function of laser fluence. We do not observe any threshold behavior here as it is expected for a doped bulk semiconductor[11;12;13;14].

References

[1] Holldack, K. *et al.* FemtoSpeX: a versatile optical pump-soft X-ray probe facility with 100 fs X-ray pulses of variable polarization. *J. Synchrotron Rad.* 1–15 (2014).

[2] Ovsyannikov, R. *et al.* Principles and operation of a new type of electron spectrometer - ArTOF. *Journal of Electron Spectroscopy and Related Phenomena* **191**, 92–103 (2013).

[3] Kühn, D. *et al.* Capabilities of Angle Resolved Time of Flight electron spectroscopy with the 60° wide angle acceptance lens. *Journal of Electron Spectroscopy and Related Phenomena* **224**, 45–50 (2018).

[4] Holldack, K. *et al.* Single bunch X-ray pulses on demand from a multi-bunch synchrotron radiation source. *Nature Communications* **5** (2014).

[5] Lu, C.-P., Li, G., Watanabe, K., Taniguchi, T. & Andrei, E. Y. Mos 2: choice substrate for accessing and tuning the electronic properties of graphene. *Physical review letters* **113**, 156804 (2014).

[6] Belete, M. *et al.* Dielectric properties and ion transport in layered mos2 grown by vapor-phase sulfurization for potential applications in nanoelectronics. *ACS Applied Nano Materials* **1**, 6197–6204 (2018).

[7] Rasmussen, F. A. & Thygesen, K. S. Computational 2d materials database: electronic structure of transition-metal dichalcogenides and oxides. *The Journal of Physical Chemistry C* **119**, 13169–13183 (2015).

[8] Beal, A. R., Hughes, H. P. & Liang, W. Y. The reflectivity spectra of some group VA transition metal dichalcogenides. *Journal of Physics C: Solid State Physics* **8**, 4236–4234 (2001).

[9] Wavemetrics, I. IGOR Pro 7. *URL: http://wavemetrics.com* .

[10] Kukk, E. Curve fitting macro package SPANCF. *URL: http://www.geocities.com/ekukk* .

[11] Widdra, W. *et al.* Time-resolved core level photoemission: surface photovoltage dynamics of the SiO_2/Si (100) interface. *Surface Science* **543**, 87–94 (2003).

[12] Bröcker, D., Gießel, T. & Widdra, W. Charge carrier dynamics at the SiO_2/Si(100) surface: a time-resolved photoemission study with combined laser and synchrotron radiation. *Chemical Physics* **299**, 247–251 (2004).

[13] Siffalovic, P., Drescher, M. & Heinzmann, U. Femtosecond time-resolved core-level photoelectron spectroscopy tracking surface photovoltage transients on p-GaAs. *EPL (Europhysics Letters)* **60**, 924 (2002).

[14] Neppl, S. *et al.* Capturing interfacial photoelectrochemical dynamics with picosecond time-resolved X-ray photoelectron spectroscopy. *Faraday discussions* **171**, 219–241 (2014).
